# Occurrence of Psoriatic Arthritis during Interferon Beta 1a Treatment for Multiple Sclerosis

**DOI:** 10.1155/2014/949317

**Published:** 2014-04-15

**Authors:** Éric Toussirot, Matthieu Béreau, Marie Bossert, Imad Malkoun, Anne Lohse

**Affiliations:** ^1^Clinical Investigation Center Biotherapy INSERM CIC-1431, FHU INCREASE, University Hospital Besançon, Place Saint Jacques, 25000 Besançon, France; ^2^Department of Rheumatology, University Hospital Besançon, 25000 Besançon, France; ^3^Department of Therapeutics, University of Franche-Comté, 25000 Besançon, France; ^4^Pathogens and Inflammation Laboratory, University of Franche-Comté, UPRES EA 4266, SFR FED 4234, 25000 Besançon, France; ^5^Department of Neurology, University Hospital Besançon, 25000 Besançon, France; ^6^Department of Rheumatology, Belfort-Montbéliard Hospital, 90000 Belfort, France; ^7^Department of Neurology, Belfort-Montbéliard Hospital, 90000 Belfort, France

## Abstract

Interferon beta (IFN-**β**) is the first line therapy of relapsing-remitting multiple sclerosis. IFN-**β** is a cytokine that can contribute to the development of systemic autoimmune disease including psoriasis. The development or the exacerbation of psoriasis during IFN-**β** treatment has been previously observed. We report the occurrence of arthritis and dactylitis in a multiple sclerosis patient with preexisting psoriasis diagnosed as a psoriatic arthritis. The IL-23/Th17 pathway is involved in psoriasis and psoriatic arthritis and it has been suggested that IFN-**β** therapy in patients with Th17-mediated disease may be detrimental. Together with previous similar reports, our case suggests that IFN-**β** should certainly be used with caution in patients with concomitant systemic autoimmune disease with IL-23/Th17 involvement.

## 1. Introduction

Interferon beta (IFN-*β*) is the first line therapy for relapsing-remitting multiple sclerosis (MS). The most common side effects of IFN-*β* include flu-like symptoms, cutaneous reactions at the injection site, and liver damage [1]. IFN-*β* is a cytokine that can contribute to the pathogenesis of different autoimmune diseases, such as systemic lupus erythematosus and rheumatoid arthritis (RA). However, type I interferons (IFNs) are also important mediators of inflammation in psoriasis. Thus, IFN-*β* therapy can potentially favour the development of systemic autoimmune diseases. We report here the occurrence of articular symptoms related to psoriatic arthritis (PsA) in a patient with MS and psoriasis receiving IFN-*β*.

## 2. Case Presentation

**Figure 1:**
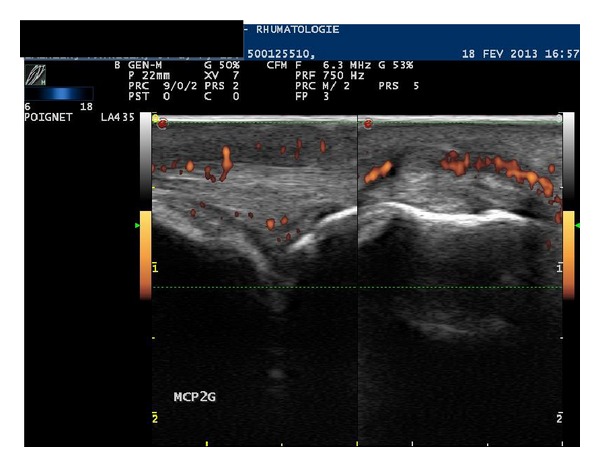
Active synovitis and dorsal tenosynovitis of the second left metacarpophalangeal joint in a 54-year-old woman with relapsing-remitting multiple sclerosis treated by IFN-*β* 1a.

The patient was a 54-year-old woman with relapsing-remitting MS diagnosed in 2004 after attacks of cerebellar syndrome and sphincter disorders. She was treated by IFN-*β* 1a, first Rebif (Merck Serono, Lyon, France) for 6 years and then Avonex (Biogen Idec, Nanterre, France). This treatment was well tolerated and the patient remained relapse-free. She also had plaque psoriasis requiring no treatment. At the end of 2010, after 9 months of Avonex treatment, the patient developed polyarthralgia with nocturnal pain and morning stiffness involving metacarpophalangeal joints and wrists, associated with joint swelling and dactylitis. Conversely, her psoriatic lesions remained unchanged. Biological tests were negative for rheumatoid factors and anti-CPP antibodies, while C-reactive protein levels were mildly elevated (12 mg/L). Ultrasound examination of the hands found active synovitis and tenosynovitis (Figure 1) but no enthesitis. There were no structural damage on hand and foot X-rays and no sacroiliitis on pelvic X-rays. Since Avonex therapy was effective for MS, this treatment was maintained and the patient successively received methotrexate, sulfasalazine, and leflunomide without any improvement in joint symptoms. Corticosteroids were of limited efficacy and thus, hydroxychloroquine was introduced in 2013, leading to partial resolution of the arthralgia.

## 3. Discussion

This case illustrates relapsing-remitting MS well controlled by IFN-*β* 1a. Under this treatment, the patient developed oligoarticular symptoms that responded to the CASPAR criteria [2] and PsA was diagnosed. While maintaining IFN-*β* 1a treatment, the articular manifestations persisted and were partially controlled by a traditional disease modifying antirheumatic drugs (DMARDs). Exacerbation of cutaneous psoriasis has previously been reported in a limited number of patients under IFN-*β* [3, 4]. These cases included one patient with a pustular flare of quiescent psoriasis [3], similar worsening of psoriasis in a small number of patients receiving IFN-*β* 1a [4], and development of new-onset psoriasis in one case [5]. In contrast, the development of arthritis during IFN-*β* 1a treatment has rarely been observed. La Mantia and Capsoni described a patient with relapsing-remitting MS who suffered severe worsening of cutaneous psoriasis and activation of oligoarticular PsA during IFN-*β* treatment [6]. The symptoms resolved after cessation of therapy. In parallel, rare cases of RA have been reported with IFN-*β* [7].

The exacerbation or the development of psoriasis/PsA during IFN-*β* therapy raises the question of the direct role of this treatment. Psoriasis and PsA are both T-cell mediated diseases. Skin biopsies from patients receiving IFN-*β* showed strong expression of the CCL2 and CXCL10 chemokines, facilitating traffic of T cells from the circulation to the skin lesions [8]. On the other hand, the IL-23/Th17 pathway has been implicated in the pathogenesis of psoriasis and PsA. Indeed, ustekinumab, a p40 IL-12/IL-23 monoclonal antibody, has proven to be highly effective in psoriasis and PsA. It has been hypothesized that IFN-*β* therapy in patients with Th17-mediated disease may have detrimental consequences [9]. Indeed, in an experimental allergic encephalomyelitis model of MS passively induced by Th17 cells reactive to myelin antigens, IFN-*β* exacerbated disease symptoms [9]. MS patients who do not respond to IFN-*β* are characterized by high serum concentration of Th17 cytokines [10]. In addition, the therapeutic response to IFN-*β* in MS patients has been related to the direct inhibition of IL-17A [9].

Taken together, these observations suggest that IFN-*β* and Th17 may be a hazardous combination in the same patient, especially one with preexisting autoimmune disease. Conversely, psoriasis may be associated with MS, although this combination remains uncertain. Psoriatic arthritis may occur in around 30% of patients with cutaneous psoriasis and we cannot exclude in our patient the spontaneous development of PsA. Finally, the treatment of arthritis in a patient with MS well controlled by IFN-*β* may be difficult. Traditional DMARDs should be proposed as first line. TNF*α* inhibitors must be avoided since these agents may worsen neurologic symptoms in MS. Ustekinumab is not effective in MS. Dimethyl fumarate is effective for psoriasis and MS, but this compound has not been evaluated in PsA. In conclusion, clinicians must be aware that induction or exacerbation of psoriasis/PsA during IFN-*β* therapy is possible. In case of concomitant systemic autoimmune disease with IL-23/Th17 involvement, IFN-*β* should certainly be used with caution.
